# Access Is Progress: Understanding Rural Secondary Student Access and Outcomes of Advanced Placement Courses

**DOI:** 10.3390/ejihpe15070143

**Published:** 2025-07-21

**Authors:** Phillip D. Grant, Ali Jahanaray, T. Logan Arrington

**Affiliations:** 1 Department of Educational and Organizational Leadership, Clemson University, Clemson, SC 29630, USA; jahanar@g.clemson.edu; 2 Department of Educational Technology and Foundations, University of West Georgia, Carrollton, GA 30118, USA; tlarring@westga.edu

**Keywords:** rural education, advanced curriculum, college and career readiness, student persistence, academic success

## Abstract

This study examines the availability and outcomes of Advanced Placement (AP) courses in secondary schools in Georgia (USA) and South Carolina (USA), focusing on how school locale (rurality) and demographic composition influence AP availability and student achievement. The authors analyzed population-level school data from the 2021–22 academic year using a two-step quantitative approach. A zero-inflated negative binomial regression model (ZINB) was employed to assess AP course participation and AP exam performance while addressing overdispersion and excess zeros in the data. Key predictors included school locale (rural versus nonrural), state (Georgia versus South Carolina), and minoritized-majority status. This study finds that rural schools and those where minoritized students make up the majority (i.e., fewer than 50% White students) are significantly less likely to offer AP courses or have any students participate in AP exams. Moreover, these schools had a significantly lower success rate; for example, rural schools were 59% less likely to have students with scores above three. The findings indicate that gaps in access to advanced curriculum may exacerbate existing college and career readiness disparities. Moreover, this study confirms that previous research using sampled datasets underestimates the disparity of AP access.

## 1. Introduction

In the United States, students who attend rural schools are offered advanced curricular programming that yields postsecondary credit at lower rates than their nonrural peers (Byun et al., 2012b, 2017; Finn & Scanlan, 2020; Price, 2021). The curriculum offered to American students is tiered, with some students being offered courses that yield postsecondary credit. These students are typically offered one of three options: dual enrollment, the International Baccalaureate (IB) Diploma program, or Advanced Placement (AP) courses. Amongst these three options, AP courses are the most used by American secondary students, with around 35% of students taking at least one AP exam (College Board, 2024).

Despite their popularity, AP courses are not as widely available in rural schools as they are in urban and suburban areas of the US, particularly those in minoritized-majority schools (Byun et al., 2012b; Clark et al., 2022; D. R. Means et al., 2024). Minoritized-majority schools are those in the United States where less than 50% of the students identify as White (Hinnant-Crawford, 2019). This inequity was widely reported in the academic literature, as shown by Byun et al. (2012a, 2012b) National Educational Longitudinal Study of 1988 (NELS88) analyses. These foundational pieces have served as a cornerstone for academic literature about advanced curricula in rural spaces for many studies (e.g., Koricich et al., 2018; D. R. Means et al., 2016b; Sowl & Crain, 2021). However, this study was based on data from students born in 1974 who are now around 50. Moreover, the dataset used is based on a large sample of students rather than population-level data. Population assessments of AP course participation are challenging, as the College Board, the nonprofit provider of AP tests, does not publicly divulge participation data at the population level. Moreover, there is some evidence that rural and nonrural curricular gaps have narrowed in recent decades (Wells et al., 2019), so more research is needed to understand if these gaps have narrowed under some state-based policy environments.

This study is unique in treating state-based data and using Zero-Inflated Negative Binomial Regression Modeling [ZINB] (Faroughi & Ismail, 2017; Mwalili et al., 2008). The use of state-based data will be critical in the future, as the fate of federal datasets in the US is not certain. The Trump administration has signaled its intention to reduce funding for the National Center for Education Statistics (Kamper, 2025), cut the U.S. Department of Education’s staff in half (Mosley, 2025), and potentially close the Department entirely (Faguy, 2025). With these factors in mind, scholars must think dynamically about available data, including state-based datasets, to understand the impact of advanced educational programming.

### 1.1. Literature Review

This review explores advanced secondary school curricula that provide postsecondary credit, focusing on AP courses in rural education. It examines existing research on student representation in AP courses and the connection between AP participation, postsecondary success, and graduate employability.

#### 1.1.1. Advanced Curriculum

Access to advanced curriculum is critical to the success of all students for the 21st century, as the economy and political realities rapidly shift. One longitudinal study found that rural students perform more poorly on math assessments because they have limited access to advanced math courses (Irvin et al., 2017). To access an advanced curriculum in a secondary school that leads to postsecondary credit, public school students in the US tend to gravitate towards one of three options: AP, dual enrollment, or the IB Diploma program.

These options are considered the preparatory curriculum for postsecondary-bound students in the US, as they simultaneously provide course credit in secondary education and at the postsecondary level (Çetin et al., 2014). Students in these programs earn credit for their postsecondary bachelor’s degree education if they are successful in passing the course (dual enrollment), completing the program (IB), or passing a standardized test (AP). Due to economies of scale, IB programs are expensive and difficult to maintain in rural areas (Thier & Beach, 2021; Meadows, 2017). Dual enrollment courses offer students the opportunity to earn both high school and college credit simultaneously by taking classes at technical schools, colleges, or universities (Cain, 2021). However, schools that serve primarily minoritized students tend to offer dual enrollment courses less often than those that do not (Spencer & Maldonado, 2021). Finally, the College Board provides AP teachers with established standards to prepare students for a standardized exam, which offers the opportunity to earn postsecondary credit for a fee of USD 98 per exam attempt. Students can retake the exam if they do not pass or want to improve their score, but the test is only offered once per year, and so it is not often retaken.

After completing an AP course in their secondary schools, American students receive a standardized score of one to five; they earn postsecondary credit with grades that span from A+ to C at the discretion of the accepting institution (College Board, 2024). Scores of three result in a C, C+, or B− depending on the postsecondary institution’s policy, while fours result in a B, B+, or A−. The highest score, five, always results in an A or A+.

If offered in their secondary school, students have the option to take AP, dual enrollment, or IB in any combination they choose. In rural schools, however, there is often only one choice. Nationally, there may be significant overlap between students who take AP and dual enrollment credit; no current study can substantiate such a claim, however (Ryu et al., 2024).

Combined, these three options have benefits and detriments. IB programs are difficult to create and maintain in rural areas, as they require expensive professional development and additional resources small schools do not possess (Meadows, 2017; Thier & Beach, 2021). Dual enrollment is an excellent option for rural students; however, it comes with challenges, such as requiring students to travel up to 30 miles to a postsecondary institution after school, often without access to public transportation (Zinth, 2014). Virtual dual enrollment courses are an option, but not all students can access high-speed internet in their homes. Moreover, at least two studies have highlighted how power imbalances between postsecondary institutions and rural schools often complicate dual enrollment agreements for the latter (Hornbeck et al., 2023; Howley et al., 2013). Although these power imbalances exist, rural schools are more likely to offer dual enrollment courses than AP (Clayton, 2021). Rural schools should choose the option that best serves their students, but the literature concerning rural administrative decision-making concerning advanced curriculum is shallow (Roberts & Grant, 2021; VanTassel-Baska & Hubbard, 2015).

#### 1.1.2. AP for Rural Education

AP courses are a unique option to provide postsecondary credit for their students without the power imbalance of dual enrollment and the heavy expense of offering the IB Diploma program. Teachers in rural areas seeking to offer AP courses need additional support, including updated textbooks aligned with AP exam standards and specialized professional development, such as training provided by an AP Summer Institute (The University of Georgia Center for Continuing Education & Hotel, n.d.). While these expenses are new, they are minimal compared to the costs of establishing the IB Diploma program or covering dual enrollment tuition in states where free tuition for dual enrollment students is not provided. Moreover, AP courses can be taught with existing teachers within the school. Thus, AP courses offer postsecondary credit pathways that may be more practical for some rural secondary schools than IB or dual enrollment programs.

Despite their benefits in offering low-cost postsecondary credit to rural students, AP courses appear to be less accessible in rural schools compared to their nonrural counterparts (Byun et al., 2012b, 2017; Finn & Scanlan, 2020; Morton et al., 2018; Price, 2021). This lack of access has led rural students in the US to attend less selective institutions than what they academically qualify for; one study found that rural students were more likely to attend a less selective postsecondary institution than their urban and suburban peers because of this lack of access (Byun et al., 2015). Among those sampled in the study, 69% of rural students reported access to an AP course compared to 96% of suburban and 93% of urban students. Similarly, a study using data from the Rural High School Aspirations Study (*n* = 2112) collected in the 2007–08 academic year found that only 19% of students sampled reported having access to any college preparation activities, including taking an AP course (Byun et al., 2017). These studies show that rural students have little access to AP courses. In STEM, AP courses may be even less available, as an emerging study found that 52.5% of secondary schools in the US offered AP Calculus AB, while only 35.3% offered AP Statistics. However, rural schools were much less likely to offer these courses, as 27.2% and 20% offered Calculus AB and Statistics, respectively (Boles, 2024).

The ongoing teacher shortage in rural areas of the United States makes it difficult for school districts to provide advanced coursework (Ingersoll & Tran, 2023). As a result, some rural schools are seeking online options in providing AP courses to students. One study of rural students’ curricular experiences found that virtual AP courses improved AP participation without sacrificing success, as students were slightly more likely to pass the AP exam than those rural students who had taken the course in person (LeBeau et al., 2020). Indeed, virtual courses are central to providing diverse educational opportunities to rural students (VanTassel-Baska & Hubbard, 2015). Moreover, providing teachers with the autonomy to teach an AP course could improve the morale of teachers who are difficult to recruit in rural areas (Ingersoll & Tran, 2023).

#### 1.1.3. AP Representation

Like other programs that allow students to earn postsecondary credit as part of their secondary curriculum, representation by minoritized students in AP participation is of concern. One analysis from the Civil Rights Data Collection (*n* = 20,000) found that White students are 16% more likely to participate in AP courses than Black students and 10% more likely than those who identify as Hispanic (Xu et al., 2021). These gaps were less pronounced in rural schools than in urban and suburban areas. Thus, AP expansion in rural areas might not suffer from racial and socioeconomic gaps compared to their nonrural counterparts. However, at least one study has found that expanding AP courses disproportionally benefits students from high-income backgrounds and those who identify as White (Owen, 2021). Though these gaps in participation in AP exist, some gaps are closing as the participation of Black students on AP exams rose by 200% from 1994 to 2013 (Kolluri, 2018).

To solve the widening participation gaps and to provide students with an advanced curriculum in their secondary education, some states mandated offering at least one AP course, starting with South Carolina in 1984 (South Carolina Department of Education, 2024). A policy analysis of states that mandate schools to offer at least one AP course found that the likelihood of graduates taking at least one AP exam increased by around 5% (Callen & Stoddard, 2024). However, the study relied on states having de jure policies, even if they were not actively enforced. For example, according to our analysis, South Carolina, the first state to mandate AP access, had 64 out of 249 high schools (26%) with no students taking AP exams during the 2021–22 academic year (South Carolina Department of Education, 2024). Therefore, it is unclear which states are actively enforcing rules that require secondary schools to offer an advanced curriculum to their students (B. Bagley, personal communication, 17 April 2025). Another study found that universal access to AP courses did not improve enrollment by low-income, English Language Learners, or students with an individualized education plan (Parra-Martinez et al., 2025). Therefore, mandating that schools offer AP courses may not be enough to drive equity efforts in public schools.

Unlike South Carolina, Georgia does not have a law in place that mandates offering at least one AP course. However, Georgia has a history of prioritizing AP courses as illustrated through various government documents, including its Race to the Top application to the Obama administration, which laid out AP as a pillar of its reform agenda, including one AP test fee for all Georgia public school students (GOSA, 2010). Ultimately, Georgia was awarded a Race to the Top grant, leading to a $700 million innovation fund for various projects, including expanding AP efforts (Cavanagh, 2011). South Carolina, however, chose to drop out of the grant competition in the second round (Smith, 2011).

More recently, both states received an influx of COVID-19 relief money, though it is unclear how this funding impacted AP course delivery. Is it likely that at least some of this funding in Georgia went to teacher training for AP courses (Tagami, 2021). Legislators from both states have been at odds with the College Board over the past few years. In 2015, Georgia legislators threatened to cancel AP US History, the College Board’s most popular AP offering for its students (Downey, 2015). Then in 2024, Georgia issued a policy that state funding could not be used to pay for the AP African American Studies test fee, which was eventually reversed after public outcry (Young, 2024). South Carolina also eliminated AP African American Studies in its high schools, but without a reversal (Alfonseca, 2024). Thus, both states have had a contentious relationship with the nonprofit provider of AP, the College Board. However, both states rely heavily on the College Board to provide postsecondary curriculum to their public school students. If the Trump administration continues to divert federal funding from education, expansion of AP efforts must likely occur at the state level.

#### 1.1.4. AP Outcomes

Student performance on AP exams is not well understood. Some studies track AP exam results and how they relate to outcomes, such as the likelihood of completing graduate school (Bleske-Rechek et al., 2004; Parra-Martinez et al., 2025) or seeking economics as a college major (Ahlstrom, 2021). One study found that American students’ overall achievement on AP exams declined from 66% to 59% from 1992 to 2012 (Judson & Hobson, 2015; Judson, 2017). During that same time, Black, Hispanic, and Native American students had declining passing rates (Palermo et al., 2022). At least one study found that rural students do not pass AP exams as frequently as their nonrural peers (Gagnon & Mattingly, 2016).

As of 2019, about a third of American secondary students took a course that resulted in postsecondary credit (National Center for Education Statistics, 2019). Scholars have good reason to believe that those participating in courses that yield postsecondary credit will result in greater employability after their formal education ends. For example, participation in AP has been linked with success in postsecondary institutions and employability through postsecondary entrance exam scores (Ackerman et al., 2013; Chatterji et al., 2021; Mo et al., 2011; Warne, 2017), graduation rates (Ackerman et al., 2013; Klopfenstein, 2010; Owen, 2021), and rates of obtaining advanced degrees (Bleske-Rechek et al., 2004; Parra-Martinez et al., 2025).

Higher rates of AP participation might also influence STEM career choices. For example, a study of rural young people in India found that those with higher self-efficacy beliefs were more hopeful regarding their educational future (Irdam et al., 2025). One possible way to increase confidence might be increased access at remote schools, where America lags behind other nations in having students take AP STEM courses. This could potentially make them more employable, especially for those interested in STEM areas that promote a lot of telecommuting positions. In turn, remote work could serve as a powerful economic driver for revitalizing rural communities in the United States. Additionally, a study of the NELS88 dataset found that curriculum intensity was a predictor of bachelor’s degree attainment and that rural students’ lack of access to AP and other advanced courses was leading to lower completion rates compared to their nonrural peers (Byun et al., 2012a).

High-achieving students expect their formal education to provide all the tools they need for success in their careers (Lock & Kelly, 2022). However, secondary students also report that what they learn today will not be relevant to their future jobs, as the skills necessary for the present and future job market are uncertain with the advent of generative artificial intelligence (Seevaratnam et al., 2023). AP is, therefore, critical to graduate employability, as these high-achieving students will need to construct their careers with uncertainty at every stage.

#### 1.1.5. Rural AP Performance

Several studies have examined rural student performance in AP exams using longitudinal and nationally sampled data. Using data from students who graduated high school in 1988, Byun et al. (2015) found that rural students were less likely to pass an AP exam than their suburban peers even after controlling for socioeconomic status, indicating a systemic barrier to success. A later analysis using data from the secondary Class of 2002 found the same pattern (Koricich et al., 2018). However, access may drive success, as at least one study has shown that rural students are successful on AP exams when offered resources that are comparable to what is offered in suburban and urban schools (B. Means et al., 2016a).

## 2. Materials and Methods

The statistical analysis was conducted using the following software and versions: R (Auckland, AU) version 4.4.1, utilizing the {zeroinfl()} function from the {pscl} package and the {glm.nb()} function from the MASS package and JMP Pro (Cary, NC, USA) version 18 for descriptive statistics and visualizations. Statistical comparisons in Table 1 and Table 2 showed that ZINB provided the best fit for the data, especially given the high number of zeros and overdispersion in the data.

### 2.1. Purpose and Research Questions

This study aims to assess the availability of AP courses in rural schools and the success of these courses in public schools in the US states of Georgia and South Carolina using population-level data. While some research has explored AP access for rural students through qualitative studies (Grant, 2022) and longitudinal analyses (Gagnon & Mattingly, 2016), population-level assessments remain scarce. For decades, rural scholars have called for national datasets to more adequately sample rural youth (Grace et al., 2006). Other fields have also noted rural under-sampling as a significant challenge in understanding rural people. For example, one study of medical prevention suggests that longitudinal datasets should oversample rural areas so that rural people are adequately included (Zahnd et al., 2019). Moreover, rural people tend to be more difficult to follow up with throughout their participation, as the infrastructure for communication is less reliable (David et al., 2013). In education research, rural participants tend to cease their participation in longitudinal studies more often than nonrural participants. For example, the custodians of the Education Longitudinal Study of 2002, which is often used to assess AP participation and success, collapsed some variables due to declining participation (Ingels et al., 2007; Xu et al., 2021). Therefore, studies of rural people that use population-level data should be more accurate and precise than longitudinal datasets.

Some studies of AP access use the Civil Rights Data Collection, which offers summarized rather than raw data (Price, 2021). As with other federally funded datasets, the Civil Rights Data Collection may cease operations under the current administration (Frankenberg & Gopalan, 2025). Georgia and South Carolina were chosen for this analysis because they are two of the few states offering school data on AP participation and pass rates by school and individual tests, which is not available in the Civil Rights Data Collection (South Carolina Department of Education, 2024; GOSA, n.d.). This unique opportunity to assess AP participation and success rates at the population level allowed us to understand relationships between AP test participation and performance, geography (rurality and state), and minoritized-majority status. We sought to understand how these categorical variables interact with and predict AP participation and pass rates. This study seeks to answer the following research questions:

1. Is there a relationship between AP test participation, locale, state, and minority status in Georgia and South Carolina?
2. Is there a relationship between AP scores above three, locale, state, and minority status in Georgia and South Carolina?

### 2.2. Data Description

This study analyzed data in several categories, including the number of students taking AP tests and the number of students scoring a three or higher from state government sources (GOSA, n.d.; South Carolina Department of Education, 2024). AP records and data are assessed and collected by the College Board in a consistent multi-step process across the US. NCES uses the US Census Bureau to develop school codes (including rurality) from the school’s geographical location, which is consistent for all states.

We collected and cleaned raw data from multiple sources (see Appendix A), standardizing labels and aligning variables across datasets. Afterward, we merged the datasets using unique school IDs and handled missing values through multivariate imputation. The final dataset combined different datasets from the state and federal levels, including the Common Core of Data [CCD] (National Center for Education Statistics, n.d.). CCD data included NCES geographical variables (based on state and school locale), and racial composition of schools (majority-minoritized). We included the raw data sources in Appendix A.

In the reported data by the South Carolina and Georgia Departments of Education in 2021–2022 (the latest data to compare states in the same year), there were 481 public secondary schools in Georgia and 256 in South Carolina. Among them, 458 schools were categorized as nonrural high schools based on NCES codes, and 279 were rural high schools. Rural schools in this study are defined in alignment with the NCES codes 41-Rural: Fringe, 42-Rural: Distant, and 43-Rural: Remote. The remaining NCES codes were considered nonrural. We chose this definition of rurality as it is the most used in rural education research in the United States (Thier et al., 2021; Longhurst, 2021). The use of NCES codes allowed us to compare schools across state lines.

### 2.3. Model Selection

Initially, the study used the Quasi-Poison Regression Model (QPRM) as a starting point for analysis of the type of data we had, as suggested by Fogarty (2023), as we observed that the data variance was much larger than our mean, resulting in overdispersion in count data. In Table 1, the QPRM provided some results; however, its model fit is weak and unreliable. There is a very large residual deviance, AIC, and it could not handle both overdispersion and excess zeros. NBRM produced acceptable results in lower residual deviance, AIC, and convergence iterations than QPRM. Moreover, NBRM handled overdispersion but not the excess zeros. ZI-NBRM, on the other hand, handled both overdispersion and excess zeros. It has the lowest residual deviance, AIC, which suggests a stronger fit. A further comparison between ZI-NBRM and NBRM is shown in Table 2.

### 2.4. Zero-Inflated Regression Model

Zero-inflated regression consists of two processes: a logistic regression to model structural zeros and a negative binomial regression to model positive counts in the data.

$$\mathrm{Binary}\;\mathrm{Process}:\;\log(\frac{\pi_{i}}{1-\;\pi_{i}})=\;\gamma_{0}+\gamma_{1}Z_{1i}+\;\gamma_{2}Z_{2i}+\cdots +\;\gamma_{q}Z_{qi}$$

The model uses the probability of structural zeros ($\pi_{i})$ and $Z_{1i},\;Z_{2i},\ldots ,Z_{qi}\;is,$ variables as predictors. The binary process (γ) produces coefficients to determine how predictors influence the probability of arising zeros from the model.

$$\mathrm{Count}\;\mathrm{Process}:\;\log\left(\mu_{i}\right)=\;\beta_{0}+\;\beta_{1}X_{1i}+\;\beta_{2}X_{2i}+\cdots +\;\beta_{p}X_{pi}\;P_{NB}\left(y\right)=\;\frac{\Gamma \left(y+\;\alpha^{-1}\right)}{\Gamma \left(\alpha^{-1}\right)\;\Gamma \left(y+1\right)}\;{\left(\frac{\alpha^{-1}}{\alpha^{-1}\;+\;\mu_{i}\;}\right)}^{\alpha^{-1}}{\left(\frac{\alpha^{-1}}{\alpha^{-1}\;+\;\mu_{i}\;}\right)}^{\;\mu_{i}}$$

α: dispersion parameter (α>0).
Γ: gamma function

The count process of ZI is assessed through negative binomial regression, except that here $\mu_{i}$ the expected positive (non-zero) count of the model is based on each predictor ($X_{1i},\;X_{2i}$… $X_{pi}$) influence coefficient ($\beta_{0},\;\beta_{1},\ldots ,\beta_{p}$). The probability of observing y under negative binomial regression is also calculated through $P_{NB}\left(y\right)$. Once both models had concluded their analysis, the final ZI looks like

$$P\;\left(Y_{i}=y\right)=\;\{\begin{array}{c} \pi_{i}\;.P_{NB}\left(0\right)\;\;\;\;\;if\;y=0,\\ \left(1-\pi_{i}\right)\;.\;P_{NB}\left(y\right)\;\;\;if\;y>0, \end{array}$$

The zero-inflated Negative Binomial regression model combines those two processes into a single process $\left(Y_{i}=y\right)$ to handle count data with overdispersion and excess zeros.

## 3. Results

The results of this study are presented in two parts. In the first part, the AP participation rate is assessed using ZINB. In the second part, AP Test Success is assessed using NBRM. In the primary analysis, the study used the total enrolment of each school for grades 9 to 12 as an offset to account for school size. Using an offset, we controlled the population of schools in the results.

### 3.1. AP Participation Rate

In the main analysis, each predictor maintained a consistent reference group: South Carolina served as the reference for the State, nonrural schools for the Locale, and minoritized-majority schools for the Minoritized predictor. In South Carolina, the mean number of AP students tested per secondary school was 114, while in Georgia, the number is 165. In rural schools, including schools with no participation, the proportion of students participating in AP tests is 0.07 (7% of students) compared to 0.12 (12% of students) of their nonrural counterparts. Thus, even when rural schools offer AP courses, fewer students participate than nonrural students.

### 3.2. Testing Regression Fit

This study aims to calculate whether the difference between rural and nonrural students’ participation in AP courses is statistically proven. To decide the best model to handle overdispersion and excess zeros, the study used Vuong’s test before running the main analysis to compare the NBRM and ZI-NBRM fit.

Table 2 shows that in all cases, Model 1 (ZI-NRBM) is statistically better than Model 2 (NBRM). Tests of significance (*p*-value < 0.0001 and z-score) show that we have strong evidence that (ZI-NRBM) is the best model fit for this study because it can account for excess zeros and overdispersion more effectively.

### 3.3. ZI-NBRM to Assess AP Availability

In Table 3, the intercept estimate (5.801, *p* < 0.001) represents the log count of AP test participation for rural South Carolina schools with a minoritized population. This baseline value suggests a significant starting point for AP participation in the reference group. The study did not consider the interactions between the predictors due to a lack of significance.

The results in Table 2 show that being in a rural school significantly lowers AP test participation for students with an intercept of −0.606, worse than the first model (*p*-value < 0.01). Except for the Locale, no other variable significantly impacts AP test participation. We are 95% confident that the actual value of the rural intercept is between (−0.80 and −0.41).

The baseline number of students participating in AP exams in nonrural, nonminoritized schools in Georgia is estimated to be approximately 330 (e^5.801^). In the count model, which estimates the expected number of participants, rural schools had significantly lower AP participation, about 45% lower than nonrural schools (e^−0.606^ ≈ 0.545, *p* < 0.001). This means rural schools have around 150 fewer students in AP exams, 180 AP test takers versus 330 students in nonrural schools. Similarly, schools in South Carolina had participation rates approximately 35% lower than schools in Georgia (e^−0.431^ ≈ 0.65, *p* < 0.001). This difference shows, for example, a Georgia school with 300 AP students might only have 195 in South Carolina. Lastly, minoritized-majority schools participated at about 73% of the rate of their nonminoritized counterparts (e^−0.306^ ≈ 0.73, *p* = 0.0015). In other words, nonwhite majority schools have 27% fewer students in AP exams. These interpretations are when we hold other variables constant.

The results for the zero-inflated component of the model that predicts the probability of a school having zero students take AP tests found rural schools (*p* = 0.005) and minoritized-majority schools (*p* = 0.020) to be more likely to have zero participation. This suggests that in addition to having lower mean participation, these schools have a greater likelihood of absolute exclusion from AP testing. These results underscore how school location, state context, and racial composition intersect to condition access to college-level coursework.

### 3.4. ZI-NBRM to Assess AP Scores Above Three

In the next part, the study aims to evaluate the success rate of students in AP tests across rural and nonrural schools. With Voung’s test, ZI-NBRM produced a better fit with AP scores above 3 as the response variable. The score of “3” was chosen as it is the minimum score examinees need to earn postsecondary credit (College Board, 2024). Reference groups: Locale (non-rural), State (Georgia), and Minoritized (White nonmajority).

The negative binomial part (positive counts) in Table 3 shows that students scoring three or above in AP tests in rural schools have significantly lower performance than nonrural schools. Rural students are expected to have 59% scores above 3, accounting for their lower success rate than nonrural schools (e^−0.90^ ≈ 0.41, *p* < 0.001). For example, if a nonrural school has 100 students who scored above 3 in AP exams, another school with the same population would achieve only 41 students scoring above three in AP exams.

South Carolina schools also performed significantly less than those in Georgia, with about 24 percent fewer students scoring above 3 on average (e^−0.28^ ≈ 0.76, *p* = 0.04). Schools with the most minoritized students also showed 39 percent lower AP performance than nonminoritized schools (e^−0.49^ ≈ 0.61, *p* = 0.0015).

The zero-inflated portion of the model in Table 4, which estimates the likelihood of having no students score above 3, showed that rural schools (*p* = 0.005), South Carolina schools (*p* = 0.02), and minoritized-majority schools (*p* = 0.001) were significantly more likely to fall into the structural zero group. Simply put, these schools (rural, minoritized-majority, and South Carolina) have fewer students scoring above 3 in AP exams and are more likely to have no students scoring above 3 compared to their peers. These results expose persistent differences in AP test outcomes shaped by geography, state context, and demographic composition of the school.

**Figure 1:**
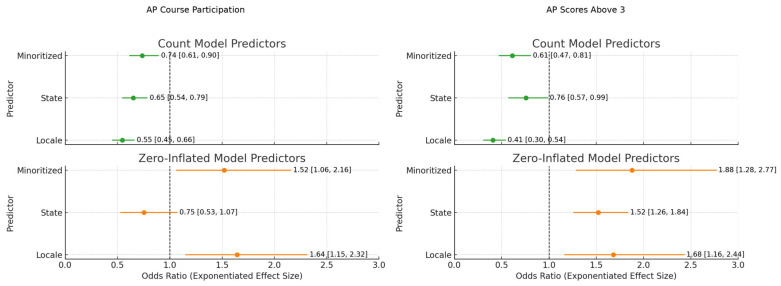
Predictors of AP Course Participation and AP Exam Success: Exponentiated Effect Sizes and 95% Confidence Intervals from Count and Zero-Inflated Models.

Figure 1 showcases the exponentiated coefficients (rate ratios and odds ratios) from Table 2 and Table 3, where ZINB assessed AP participation and AP scores above three. To the left of the vertical line, estimates less than one shows a reduced likelihood of AP participation and success based on each predictor (locale, state, majority-minoritized). Error bars represent 95% confidence intervals, and model components are color-coded for clarity.

## 4. Discussion

This study found that rural schools in Georgia and South Carolina are 64% more likely to have no students taking an AP course than in nonrural schools, similar to Gagnon and Mattingly’s (2016) estimate; they found that town, suburban, and urban districts were 27%, 42.4%, and 45.9% more likely to offer an AP course. Our study shows that rural students in Georgia and South Carolina are much less likely to have access to an AP course than nationally sampled analyses. We also found that South Carolina students participate in AP courses significantly less frequently than in Georgia. Though it is difficult to ascertain why South Carolina schools are less likely to offer AP courses, especially considering the legal mandate in South Carolina. State policymaking in South Carolina since the Obama administration could be to blame (Cavanagh, 2011; Smith, 2011; Tagami, 2021). While South Carolina mandates that each high school offer an AP course, this law has not been enforced in many years as rural schools, in particular, struggle with the ongoing teacher shortage crisis (B. Bagley, personal communication, 11 April 2025). South Carolina is experiencing a teacher shortage of around 1600 teachers, while Georgia does not publish vacancy data (South Carolina Department of Education, 2025). Georgia employs 121,301 teachers, while South Carolina employs 55,947 teachers (National Center for Education Statistics, 2022). However, in South Carolina, 14% of all classes are taught by teachers teaching out of the field (Public Education Partners, 2024). For high-poverty schools, this proportion grows to 21%. Thus, understanding why Georgia and South Carolina have a difference in their AP course offerings requires more qualitative work beyond the scope of this study. The current study is the first to assess differences in AP access in these states.

This study uniquely contributes to the literature surrounding AP schools in the US. In schools where White students are not the majority, the likelihood of offering no AP courses is 23% higher compared to schools where White students are the majority. Previous analyses of student-level data found that White students were 16% more likely to participate in AP courses than Black students and 10% more likely than those who identify as Hispanic (Xu et al., 2021). Our analysis shows that at least part of this disparity is due to structural rather than student issues; minoritized-majority schools are significantly less likely to offer AP courses, regardless of geographic locale.

Like AP test participation, rural schools have a significantly lower number of students achieving scores above three than nonrural schools. This finding is supported by Gagnon and Mattingly’s (2016) Civil Rights Data Collection analysis. In addition, students at minoritized-majority schools had significantly fewer scores above three than those who attended a primarily White school. Previous analyses that have focused on student-level data found that Black, Hispanic, and Native American-identifying students score significantly lower than White and Asian-identifying students (Judson & Hobson, 2015; Judson, 2017). Our analysis is the first to take a structural approach to understanding the school as a factor in AP performance rather than individual student performance. Future research should continue to pick apart school-level and student-level factors, as minoritized individuals and those attending a minoritized-majority school tend to pass AP exams at a lower rate.

This study examined data in Georgia and South Carolina because of the availability of population-level school-based data. In our analysis, we found that Georgia students are more likely to be offered AP courses and that they are more likely to pass the AP exam. While it is difficult to know why this statistically significant difference exists, examining policy actions over the past 15 years offers some clues. Georgia included AP in its Race to the Top application in 2010, which led to an influx of $700 million in a federal grant (GOSA, 2010). South Carolina withdrew from the competition before it concluded (Smith, 2011).

In addition to the effects of national policies, the spread of COVID-19 around the world and in the US academic community was evident. After the pandemic, students’ math and reading scores in the US dropped significantly, and the academic recovery was a burden for students (Kuhfeld et al., 2023). Moreover, both states received an influx of federal dollars in COVID-19 recovery efforts, but whether either state used this funding to expand AP access is unclear. Finally, both states have engaged in political battles with the College Board, which has resulted in South Carolina no longer offering at least one AP course (African American Studies). Georgia legislators and bureaucrats seem more amenable to the College Board’s program than South Carolina’s. Additionally, using federal dollars in Georgia helped sustain or expand AP course efforts in the state.

### 4.1. Recommendations

The finding that nonrural students are significantly more likely to pass an AP test should not be interpreted as assessing rural students’ ability. This study showed that rural students are significantly less likely to have access to a single AP course than nonrural students. Moreover, we showed that rurality is the strongest predictor of access to AP courses amongst the variables we tested (charter school status, Title I eligibility, magnet school status, and minoritized-majority status). This paucity of access for rural AP teachers means that those who do teach AP in their school may be the only AP teacher within the building, unlike those in nonrural schools.

Our findings indicate that rural schools need more support in offering and delivering AP courses in terms of both expansion and performance on AP exams, as rural students were significantly more likely not to pass an AP exam compared to their nonrural counterparts. The same interpretation can be applied to those in minoritized-majority schools. This analysis took a school-level rather than student-level approach to understand if structural factors impacted both AP availability and AP success. Our study concludes that these school factors are at play in terms of availability and access. State and school district leaders should continue coordinating efforts to offer AP courses in rural and minoritized-majority schools with individual states to avoid political theater, risking course offerings for students. To feasibly improve access to AP courses in South Carolina, the state should provide funds to districts to enforce their policy on the books that require schools to offer an AP course. However, an unfunded mandate like this could be disastrous to very small school districts.

Scholars who use federal databases in their research likely need to rely more heavily on state databases in the future. This study combined data from federal and state databases to understand AP data on a population level. This change proved difficult, as the number of zeros in the analysis inflated some estimates. Scholars should consider using ZIMB in future analyses where excess zeros are present.

### 4.2. Limitations

This study brings valuable insight with a population-level AP performance and participation analysis while acknowledging some limitations. Firstly, some covariates might influence the outcomes, such as school funding, teacher qualifications, and individual socioeconomic factors of students that were either unavailable or out of the study scope. Therefore, the absence of covariates might contribute to the omitted variable bias. Lastly, we acknowledge that our AP analysis findings may not be generalized to a broader population because other states might have different policies and structures.

## 5. Conclusions

In this study, we analyzed AP exam data in Georgia and South Carolina to understand how students at different types of schools performed. We found that rural students were significantly less likely to be offered an AP exam. Moreover, they were also significantly less likely to pass an AP exam. These findings were true for minoritized-majority schools as well. Therefore, policymakers and bureaucrats should focus on school-level efforts to expand AP course availability and exam success.

## Figures and Tables

**Table 1:** Regression Models Comparison for Suitable Count (Discrete) data.

Diagnostic Criterion	Quasi-Poisson Regression Model (QPRM)	Negative Binomial Regression Model (NBRM)	Zero-Inflated Negative Binomial Regression (ZINBR)
Dispersion Parameter (Theta)	Not Applicable	0.95 (AP Participation), 1.05 (AP Success)	1.21 (AP Participation), 1.25 (AP Success)
Null Deviance (AP Participation)	159,042	836	682
Residual Deviance (AP Participation)	119,032	723	558
Null Deviance (AP Success)	258,826	7025	6140
Residual Deviance (AP Success)	188,042	6495	5632
Akaike Information Criterion (AIC)—AP Participation	120,456	3542	3178
Akaike Information Criterion (AIC)—AP Success	196,232	7025	6140
Iterations to Convergence	6	1	2
Standard Error Estimates	Underestimated	Appropriate	Appropriate
Zero-inflation Handling	No	No	Yes

**Table 2:** Vuong’s Test Results.

	Vuong Z-Statistic	Alternative Hypothesis	*p*-Value
Raw	11.001	Model 1 > Model 2	<0.0001
AIC-corrected	10.695	Model 1 > Model 2	<0.0001
BIC-corrected	9.980	Model 1 > Model 2	<0.0001

**Table 3:** Zero-Inflated Negative Binomial Regression Model Results for AP Participation.

Count Model (NB)	Estimate	Std Error	z	*p*(sig)	95% CI
Intercept	5.801	0.094	61.726	<0.001 ***	[5.62, 5.98]
Locale	−0.606	0.991	−6.117	<0.001 ***	[−0.80, −0.41]
State	−0.431	0.095	−4.538	<0.001 ***	[−0.61, −0.24]
Minoritized	−0.306	0.096	−3.183	0.0015 **	[−0.49, −0.11]
**Zero-Inflated Model**	**Estimate**	**Std Error**	**z**	***p*(sig)**	**95% CI**
Intercept	−1.295	0.181	−7.148	<0.001 ***	[−1.65, −0.94]
Locale	0.497	0.179	2.766	0.005 **	[0.14, 0.84]
State	−0.283	0.182	−1.553	0.121	[−0.64, 0.07]
Minoritized	0.419	0.1809	2.321	0.020 *	[0.06, 0.77]

Note: *** For *p* < 0.001—Very strong evidence of statistical significance. ** For *p* < 0.01—Strong evidence of statistical significance. * For *p* < 0.05—Some evidence of statistical significance; for *p* < 0.1—No evidence of statistical significance.

**Table 4:** Zero-Inflated Negative Binomial Regression Model Results for AP Participation.

Count Model (NB)	Estimate	Std Error	z	*p*(sig)	95% CI
Intercept	6.02	0.13	45.27	<0.001 ***	[5.57, 6.28]
Locale	−0.90	0.15	−6.12	<0.001 ***	[−1.19, −0.61]
State	−0.28	0.14	−3.51	0.04 *	[−0.56, −0.01]
Minoritized	−0.49	0.13	−2.01	0.0015 ***	[−0.76, −0.21]
**Zero-Inflated Model**	**Estimate**	**Std Error**	**z**	***p*(sig)**	**95% CI**
Intercept	−1.57	0.21	−7.56	<0.001 ***	[−1.98, −1.16]
Locale	0.52	0.19	2.74	0.005 **	[0.15, 0.89]
State	0.42	0.18	3.25	0.02 *	[0.65, 0.79]
Minoritized	0.63	0.19	2.31	0.001	[0.25, 1.02]

Note: *** For *p* < 0.001—Very strong evidence of statistical significance. ** For *p* < 0.01—Strong evidence of statistical significance. * For *p* < 0.05—Some evidence of statistical significance; for *p* < 0.1—No evidence of statistical significance.

## Data Availability

The US states of Georgia and South Carolina provide AP data publicly. As of writing, the National Center for Education Statistics provides data publicly, though it has been defunded, and its future is uncertain (Appendix A).

## Abbreviations

The following abbreviations are used in this manuscript:

GOSA	Governor’s Office of Student Achievement (Georgia)
AP	Advanced Placement
IB	International Baccalaureate program
LD	Linear dichroism
NCES	National Center of Education Statistics

## Appendix A. Public Data Sources Used in the Study

**Data Source**	**Description**	**Link**
SCDE—AP Report	South Carolina AP exam performance data	https://ed.sc.gov/data/test-scores/national-assessments/ap/ (accessed on 1 January 2024)
SCDE—Enrollment	South Carolina active student headcounts	https://ed.sc.gov/data/other/student-counts/active-student-headcounts/ (accessed on 1 January 2024)
GOSA—AP and Enrollment Data	Georgia AP exam data and school enrollment statistics	https://gosa.georgia.gov/dashboards-data-report-card/downloadable-data (accessed on 1 January 2024)
NCES CCD—Locale Boundaries (EDGE)	Locale codes and geographic classification of schools	https://nces.ed.gov/programs/edge/Geographic/LocaleBoundaries (accessed on 1 January 2024)
